# Treatment of glioblastoma with tumor-specific amplitude-modulated radiofrequency electromagnetic fields

**DOI:** 10.18632/oncotarget.28770

**Published:** 2025-10-13

**Authors:** Hugo Jimenez, Denise Gibo, Sambad Sharma, Michael Pennison, Lance D. Miller, Minghui Wang, Kimberly Sheffield, Liyue Zhang, Allan Johansen, Preeya Achari, Callum Mcgrath, Sean Lester, Jason Tang, Kojo Agyemang, Annette Johnson, Christopher T. Whitlow, Michael Chan, Kounosuke Watabe, Ralph D’Agostino, Janaka Liyanage, Asfar Azmi, Geoffrey Barger, Alexandre Barbault, Glenn J. Lesser, Waldemar Debinski, Boris C. Pasche

**Affiliations:** ^1^Department of Oncology, Wayne State University School of Medicine/Karmanos Cancer Institute, Detroit, MI 48201, USA; ^2^Department of Cancer Biology, Wake Forest University School of Medicine, Winston-Salem, NC 27101, USA; ^3^Medical College of Georgia at Augusta University, Augusta, GA 30912, USA; ^4^Department of Radiology, Atrium Health Wake Forest Baptist Medical Center, Winston-Salem, NC 27103, USA; ^5^Department of Radiation Oncology, Atrium Health Wake Forest Baptist Medical Center, Winston-Salem, NC 27103, USA; ^6^Department of Neurology, Wayne State University, Detroit, MI 48202, USA; ^7^TheraBionic GmbH, Ettlingen, Germany; ^8^Department of Internal Medicine, Section on Hematology/Oncology, Atrium Health Wake Forest Baptist Medical Center, Winston-Salem, NC 27157, USA

**Keywords:** amplitude-modulated radiofrequency electromagnetic fields, glioblastoma, TheraBionic, CACNA1H, Ca_v_3.2

## Abstract

Background: Intrabuccal administration of amplitude-modulated 27.12 MHz radiofrequency electromagnetic fields (AM RF EMF) resulting in the systemic delivery of low and safe levels of AM RF EMF has shown activity in several forms of cancer.

Methods: Glioblastoma (GB) cell lines were exposed to GB-specific AM RF EMF (GBMF) three hours per day at a level of exposure identical to patients during treatment. Cellular assays and agnostic genomic approaches were used to characterize the mechanism-of-action. One patient with therapy refractory GB received compassionate use treatment with GBMF as well as a second patient with refractory oligodendroglioma.

Results: Treatment with GBMF inhibited the proliferation of several GB cell lines. CACNA1H mediates the effect of GBMF. GBMF modulates the “Mitotic Roles of Polo-Like Kinase” pathway resulting in the disruption of GB mitotic spindle. There was evidence of clinical and radiological benefit in a 38-year-old patient with recurrent GB and evidence of safety and feasibility in a 47-year-old patient with oligodendroglioma.

Conclusions: This is the first report showing *in vitro* antitumor activity, disruption of the mitotic spindle, activation of the Mitotic Roles of Polo-like kinase pathway in GB. This is also the first report showing feasibility and clinical activity in patients with brain tumor.

## INTRODUCTION

Glioblastoma (GB) remains one of the most difficult cancers to treat and is the most common malignant primary tumor of the brain [1]. Only two new treatment approaches have led to improved survival in the past decades: (1) addition of temozolomide to surgery and radiotherapy for newly diagnosed GB has increased the median survival by 2.5 months, [2] (2) addition of alternating electric fields (Tumor Treating Fields; TTFields) delivered by the Optune^®^ device has further extended survival by 4.9 months, resulting in a five year survival rate of 13% compared to 5% for the control arm [3] without negative impact on quality of life [4].

Over the past two decades we tested the hypothesis that proliferation of tumor cells can be blocked by specific frequencies. Using a patient-based approach, we exposed patients with a diagnosis of cancer to 27.12 MHz or 433 MHz radiofrequency (RF) electromagnetic fields (EMF), which are amplitude-modulated (AM) between 0.1 Hz and 100 kHz. This led us to the discovery that pulse pressure changes occur at the same modulation frequencies in patients with the same tumor type, e.g., tumor-specific modulation frequencies [5, 6]. We subsequently conducted a clinical study to determine whether delivery of these tumor-specific AM RF EMF was feasible. We designed a battery-powered, portable device connected to a coaxial cable ending with a spoon-shaped antenna placed on the patient’s tongue during treatment. Optimal absorption of RF EMF occurs at one fourth of the wavelength. We selected a carrier wave of 27.12 MHz, which has a wavelength of 11.05 meters because one fourth of the wavelength (2.75 m) is close to the size of the adult human body. Furthermore, 27.12 MHz is approved for medical use worldwide. The device emits 27.12 MHz RF EMF AMF with an output power of 100 mW into a 50 Ω (Ohm) load using a sinusoidal test signal, which results in the delivery of low levels of EMF throughout the entire body [7]. Treatment does not require hospitalization and is administered daily with a portable device for three hours, which achieves therapeutic efficacy as validated by dose response experiments. One-hour daily exposure does not result in proliferation inhibition and six-hour daily treatment does not result in greater inhibition of cancer cell proliferation than three-hour daily treatment [8, 9].

In 2023, the TheraBionic^®^ device received FDA approval for treatment of adult patients with advanced hepatocellular carcinoma who fail 1st line and 2nd line therapy. This is the first medical device approved for systemic targeted treatment of cancer. Two ongoing trials are assessing the safety and effectiveness of the device in patients with stage IV adenocarcinoma of the pancreas: NCT05776524 and NCT06576115.

While there are some end effect similarities between the TheraBionic^®^ and the Optune^®^ devices, e.g., both result in disruption of the mitotic spindle [8, 10], there are also notable differences: (1) TheraBionic^®^ is a systemic treatment delivering RF EMF at similar levels from head to toe as the whole body becomes an antenna [7] while Optune^®^ is a localized treatment restricted to the body areas around which electrodes are placed, (2) TheraBionic^®^ delivers 27.12 MHz radiofrequency electromagnetic fields, which are amplitude-modulated at multiple tumor-specific frequencies ranging from 1 Hz to 100 kHz and results in electric fields of less than 35 V/m applied three hours per day; [8] while Optune^®^ delivers alternating electric fields of 100–200 V/m (1–2 V/cm) modulated at a single frequency ranging from 100–300 kHz applied 18–20 hours per day [3, 8, 10–14]. The mechanism of action for AM RF EMF in HCC and breast cancer is mediated via targeted activation of the Cav3.2 (CACNA1H) T-type voltage gated calcium channel (VGCC) in tumor cells, leading to calcium influx triggering anti-proliferative effects solely in tumor cells [7, 9]. While the AM RF EMF CACNA1H receptor transduction mechanism is identical in HCC and breast cancer, the pathway activated are different. HCC-specific modulation frequencies modulate the IP3/DAG signaling pathway [7] while breast cancer-specific frequencies activate the CAMKII/p38 MAPK signaling pathway [9]. In contrast, TTFields have been shown to activate Ca_v_ 1.2 L-type voltage-gated calcium channel (CACNA1C) in human GB cells [15].

Treatment with tumor-specific AM RF EMF as a monotherapy has been shown to yield both complete and partial responses in patients with advanced hepatocellular carcinoma treated with hepatocellular carcinoma-specific modulation frequencies, as well as in patients with stage IV breast cancer treated with breast cancer specific modulation frequencies [5, 16]. One patient with recurrent, unresectable advanced hepatocellular carcinoma had a partial response lasting more than six years [7]. Objective tumor shrinkage has been documented in the liver, bone, adrenal gland, and brain thus demonstrating systemic targeted antitumor effects [5, 9, 16]. No adverse events other than grade 1 fatigue and grade 1 mucositis have been reported in 86 patients included in two clinical trials as well as real-world patients receiving treatment post CE approval or on compassionate use [5, 16–18] One patient with stage IV thyroid cancer metastatic to the lungs enrolled in the feasibility study in August 2006 receiving daily treatments with the device for more than 16 years after treatment initiation (data on file, TheraBionic GmbH).

This work aimed to assess and characterize GB-specific AM RF EMF anti-tumor activity in brain tumor cell lines. Additionally, we show feasibility of this novel approach in two patients with treatment-refractory oligodendroglioma and Glioblastoma, respectively.

## RESULTS

### Assessment of GB-specific AM RF EMF on GB cell proliferation

**Figure 1:**
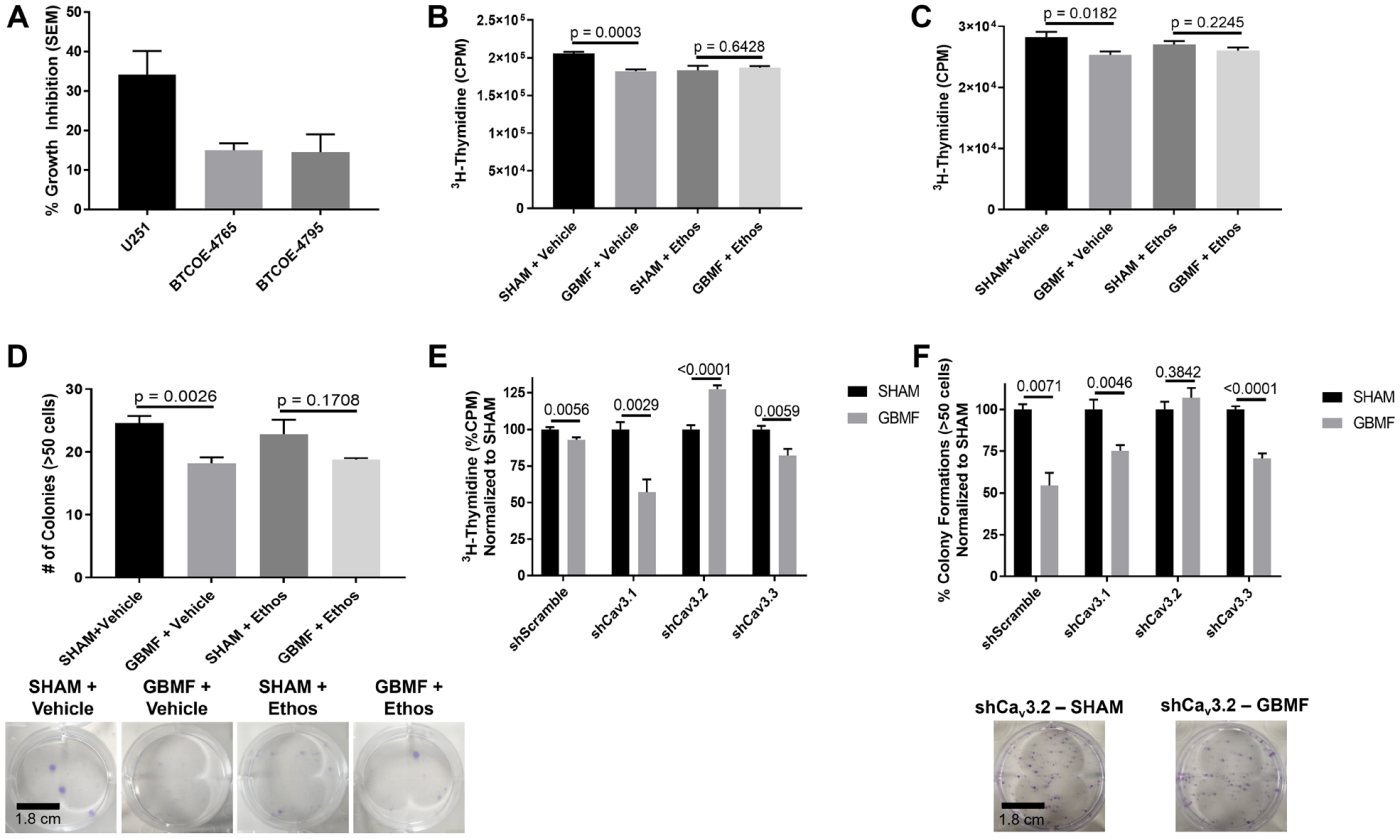
Proliferative inhibition of GB cell lines. (**A**) Tritiated thymidine incorporation assay. U251 – 34.19% Growth Inhibition (GI) with a Std Err of 6.01%; 2-tail *t*-test *p*-value 0.0001. SHAM *N* = 8, GBMF *N* = 6. BTCOE-4765 – 15.03% GI with a Std Err of 1.73%. 2-tail *t*-test *p*-value 0.0341. SHAM *N* = 6 GBMF *N* = 5. BTCOE-4795 – 14.52% GI with a Std Err of 4.48%. 2-tail *t*-test *p*-value 0.0431. SHAM *N* = 10 GBMF *N* = 10. (**B**) U251 – tritiated thymidine incorporation assay (1 mM ethosuximide) 11.5% GI GBMF treated group. SHAM+Vehicle (*N* = 5) vs. GBMF+Vehicle (*N* = 5) 2-tail *t*-test *p*-value 0.0003. SHAM+Ethos (*N* = 6) vs. GBMF+Ethos (*N* = 5) 2-tail *t*-test *p*-value 0.6428. (**C**) BTCOE-4795 – tritiated thymidine incorporation assay (1 mM ethosuximide) 10.37% GI GBMF treated group. SHAM+Vehicle (*N* = 12) vs. GBMF+Vehicle (*N* = 9) 2-tail *t*-test *p*-value 0.0182. SHAM+Ethos (*N* = 10) vs. GBMF+Ethos (*N* = 10) 2-tail *t*-test *p*-value 0.2245. (**D**) U251 – colony formation assay (1 mM ethosuximide) 26.02% Fewer colonies in the GBMF treated group. SHAM+Vehicle (*N* = 5) vs. GBMF+Vehicle (*N* = 5) 2-tail *t*-test *p*-value 0.0026. SHAM+Ethos (*N* = 5) vs. HCCMF+Ethos (*N* = 4) 2-tail *t*-test *p*-value 0.1708. (**E**) U251 (Ca_v_ Knockdowns) – tritiated thymidine incorporation assay. U251 – shScramble: SHAM *N* = 12 and GBMF *N* = 10; 2-tail *t*-test *p*-value 0.0056 – (7.01% GI). U251 – shCa_v_ 3.1: SHAM *N* = 5 and GBMF *N* = 6; 2-tail *t*-test *p*-value 0.0029 – (42.87% GI). U251 – shCa_v_3.2: SHAM *N* = 5 and GBMF *N* = 6; 2-tail *t*-test *p*-value < 0.0001 – (27.28% Growth). U251 – shCa_v_3.3: *N* = 6 per group; 2-tail *t*-test *p*-value 0.0059 – (17.8% GI). (**F**) U251 (Ca_v_ Knockdowns) – colony formation assay. U251 – shScramble *N* = 6/group, 2-tail *t*-test *p*-value 0.0071 and 45.61% fewer colonies. U251 – shCa_v_3.1 *N* = 6/group, 2-tail *t*-test *p*-value 0.0046 and 24.78% fewer colonies. U251 – shCa_v_3.2 *N* = 6/group, 2-tail *t*-test *p*-value 0.3842 and 6.88% increase in colonies. U251 – Ca_v_3.3 *N* = 6/group, 2-tail *t*-test *p*-value <0.0001 and 29.19% fewer colonies. Representative experiment shown. Experiments repeated at least twice.

The GB cell line U251, as well as three low passage (<20 passages) GB explant cells (BTCOE-4765, BTCOE-4536 and BTCOE-4795), were exposed to 27.12 MHz RF EMF amplitude-modulated at GB-specific frequencies using *in vitro* exposure systems [8] replicating levels delivered to the brain of patients during treatment with the TheraBionic^®^ device [7]. Cells were exposed daily for 3 hours for a total of 7 days for all experiments. As shown in Figure 1A, the proliferation of U251, BTCOE-4765, and BTCOE-4795 cell lines decreased by 34.19%, 15.03%, and 14.52%, respectively. The proliferation of the BTCOE-4536 cell line was not inhibited by GBMF (Data not shown). Furthermore, there was no evidence that GBMF antiproliferative effects are mediated by apoptosis (Supplementary Figures 1 and 2).

Next, we tested the hypothesis that T-type voltage-gated calcium channels (VGCC) mediate GB cell proliferation as observed in breast cancer and hepatocellular carcinoma [7, 9]. We conducted experiments in the presence or absence of ethosuximide, a pan T-type voltage-gated calcium channel (VGCC) blocker. In the presence of ethosuximide, GBMF did not block the proliferation of both U251 and BTCOE-4795 as measured by tritiated thymidine incorporation assay and colony formation assay (in U251 cells only) (Figure 1B–1D). As BTCOE-4795 did not form colonies, we performed a secondary proliferation assay, Cell titer glo, showing a 4.74% inhibition following GBMF treatment (*p* = 0.0292) (Supplementary Figure 3). To test the hypothesis that GB cell proliferation is only sensitive to GB-specific frequencies, we assessed colony formation assay with the U251 cell line and found that proliferative inhibition only occurred in response to GBMF treatment, and not to a different set of tumor-specific frequencies (HCC-specific AM RF EMF – HCCMF) (Supplementary Figure 4).

Having established that GBMF-mediated inhibition of cell proliferation depends on T-type VGCCs, we sought to determine which isoform(s) of these channels mediate this effect. We measured basal expression of T-type VGCCs in all our GB cell lines (Supplementary Figure 5). Then, we knocked down the expression of each T-type VGCC isoform: Ca_v_3.1 (CACNA1G), Ca_v_3.2 (CACNA1H), and Cav3.3 (CACNA1I) in the U251 cell line. Knockdown of all three VGCC isoforms were over 70% as quantified by qPCR. Specifically, Cav 3.1 – 79.34% knockdown, Cav 3.2 – 74.48% knockdown. Cav 3.3 – 79.60% knockdown. (Note: we were unsuccessful in our attempts to create individual knockdowns of T-type VGCCs in the BTCOE-4795).

We then assessed GBMF-mediated proliferative inhibition (Figure 1E) and colony formation (Figure 1F) in each T-type VGCC isoform knockdown. We found that Ca_v_3.2-knockdown abrogates GBMF antiproliferative effect. In contrast, knockdown of Ca_v_3.1 and Ca_v_3.3 did not affect GBMF’s antiproliferative effect.

### Cancer stem cell inhibition in GB cells

**Figure 2:**
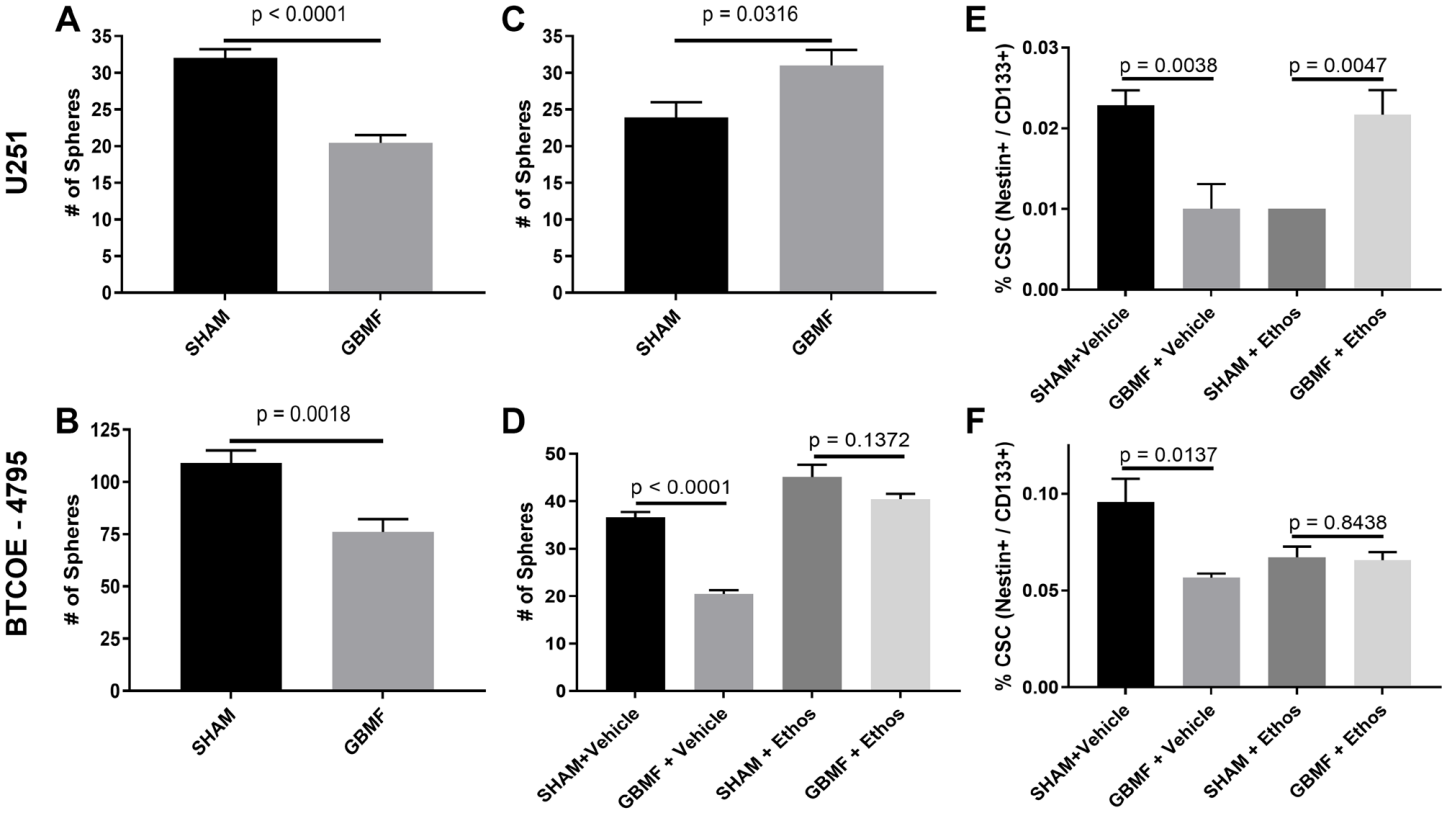
Inhibition of GB cancer stem cells. (**A**) Tumor sphere formation U251: *N* = 7/group; 2-tail *t*-test *p*-value <0.0001 – 36.16% reduction. (**B**) Tumor sphere formation BTCOE-4795: *N* = 8/group; 2-tail *t*-test *p*-value = 0.0018 – 30.16% (Increase). (**C**) Tumor sphere formation U251–shCa_v_ 3.2: *N* = 8/group; 2-tail *t*-test *p*-value = 0.0316 – 36.16% (Increase). (**D**) Tumor sphere formation BTCOE-4795 (+/− 1 mM ethosuximide) SHAM+Vehicle (*N* = 6) vs. GBMF+Vehicle (*N* = 7) 2-tail *t*-test *p*-value <0.0001 – 44.29% (Reduction). SHAM+Ethos (*N* = 8) vs. GBMF+Ethos (*N* = 7) 2-tail *t*-test *p*-value 0.1372. (**E**) Flow cytometry U251 – Cancer Stem Cells (1 mM ethosuximide) – 2 experiments performed (10,000 cells seeded and 20,000 events or greater recorded for all flow cytometry). SHAM+Vehicle (*N* = 7) vs. GBMF+Vehicle (*N* = 7) 2-tail *t*-test *p*-value 0.0038 – 56.25% (Reduction). SHAM+Ethos (*N* = 7) vs. GBMF+Ethos (*N* = 6) Mann-Whitney test *p*-value 0.0047 – 116.67% (Increase). (**F**) Flow cytometry BTCOE-4795 – Cancer Stem Cells (1 mM ethosuximide) – 2 experiments performed (60,000 cells seeded and 12,000 events or greater recorded for all flow cytometry). SHAM+Vehicle (*N* = 6) vs. GBMF+Vehicle (*N* = 7) 2-tail *t*-test *p*-value 0.0137 – 40.80% (Reduction). SHAM+Ethos (*N* = 8) vs. GBMF+Ethos (*N* = 7); 2-tail *t*-test *p*-value 0.8438. Representative experiment shown. Experiments repeated twice.

Next, we sought to determine GBMF impact on tumor stem-like cells. GBMF treatment decreased tumor sphere-forming ability of U251 and BTCOE-4795 cells by 36.16% (Figure 2A) and 30.16% (Figure 2B), respectively. To determine whether this effect is mediated by the T-Type VGCCs, specifically Ca_v_3.2, we examined the tumor sphere-forming ability of U251 with Ca_v_3.2 knockdown. There was a 29.84% increase in the number of spheres in the U251-shCa_v_3.2 cell line (Figure 2C). We were unable to knockdown Ca_v_3.2 in the BTCOE-4795 cell line. However, the growth inhibitory effect of GBMF was abolished in the presence of the T-type VGCC blocker ethosuximide (Figure 2D). Lastly, we examined the cancer stem cell population of both U251 and BTCOE-4795 by flow cytometry using two markers of stemness, Nestin and CD133. We found that the inhibitory effect of GBMF was blocked in the presence of ethosuximide (Figure 2E, 2F). We used ethosuximide treated cells for flow cytometric analysis as the U251-shCav3.2 was GFP tagged and would not allow for analysis via flow cytometry.

### Signaling pathway identification - RNA-seq and – Mitotic Roles of Polo-Like Kinase

**Figure 3:**
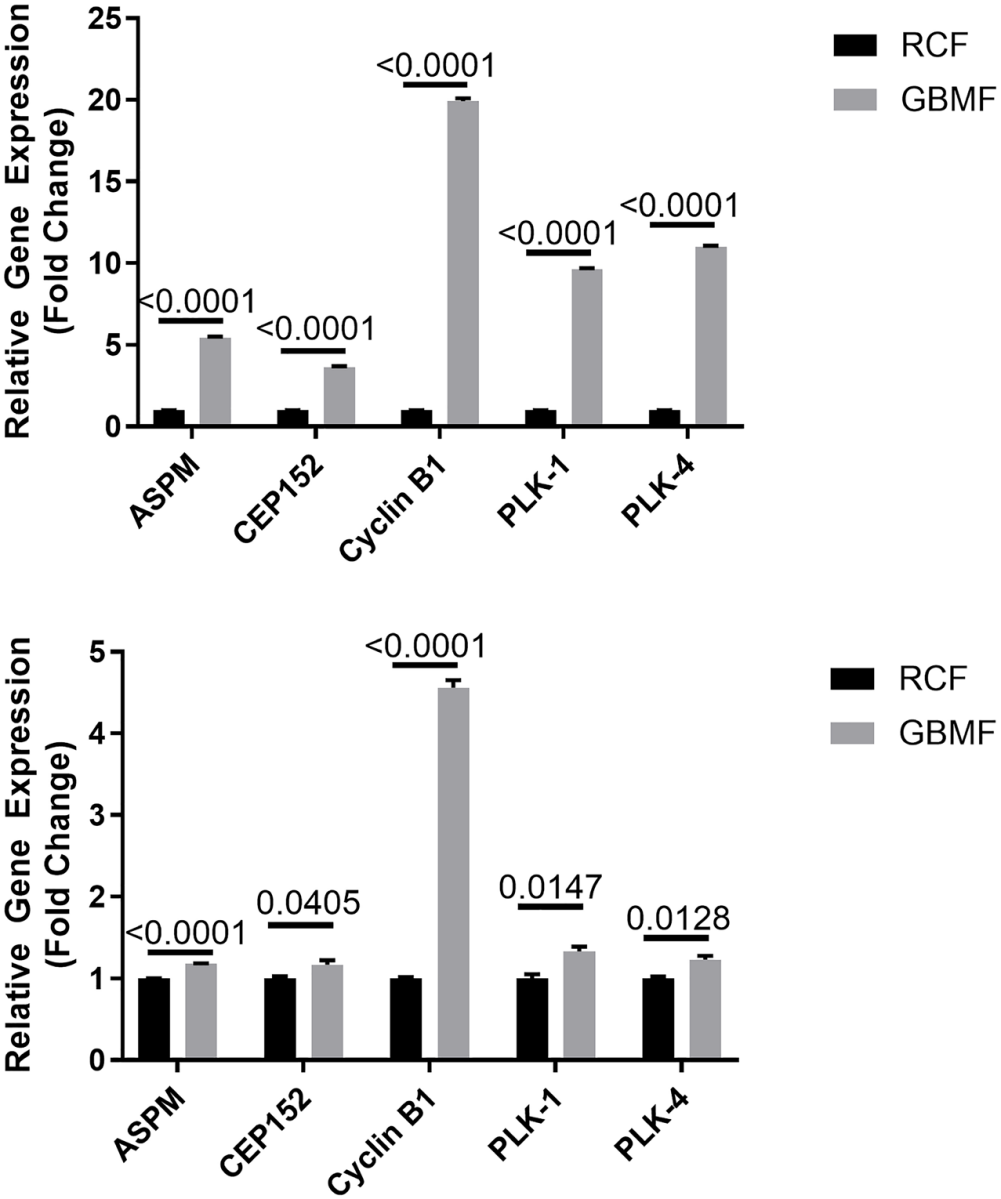
Mitotic Roles of Polo-Like Kinase. qRT-PCR validation of five differentially expressed genes from the canonical pathway Mitotic Roles of Polo-Like Kinase. qRT-PCR validation of the target genes confirmed highly significant RNA-seq differential expression. Top Figure – U251 cell line: ASPM, 5.42 fold-change increased expression (*N* = 18; 2-tail *t*-test *p* < 0.0001), CEP152, 3.62 fold-change increased expression (*N* = 18; 2-tail *t*-test *p* < 0.0001), Cyclin B1, 19.92 fold-change increased expression (*N* = 18; 2-tail *t*-test *p* < 0.0001), PLK1, 9.63 fold-change increased expression (*N* = 18; 2-tail *t*-test *p* < 0.0001), and PLK4, 11.01 fold-change increased expression (*N* = 15; 2-tail *t*-test *p* < 0.0001). Bottom Figure – BTCOE-4795 cell line: ASPM, 1.18 fold-change increased expression (*N* = 3; 1-tail *t*-test *p* < 0.0001), CEP152, 1.17 fold-change increased expression (*N* = 3; 1-tail *t*-test *p* = 0.0405), Cyclin B1, 4.56 fold-change increased expression (*N* = 3; 1-tail *t*-test *p* < 0.0001), PLK1, 1.33 fold-change increased expression (*N* = 3; 1-tail *t*-test *p* = 0.0147), and PLK4, 1.23 fold-change increased expression (*N* = 3; 1-tail *t*-test *p* = 0.0128). Representative experiment shown. Experiments repeated twice.

To agnostically identify the pathways modulated by GBMF in GB cells, we performed RNA-seq of GB U251 cells. RNA-seq data analysis followed by IPA and DAVID analysis identified the “Mitotic Roles of Polo-Like Kinase” pathway as the prime target of GB-specific AM RF EMF (Table 1). Polo-Like kinases (PLKs) are regulatory serine/threonine kinases of the cell cycle involved in mitotic entry, mitotic exit, spindle formation, cytokinesis, and meiosis [19]. Five of the differentially-expressed genes of this canonical pathway were validated by qRT-PCR: Abnormal Spindle Microtubule Assemble (ASPM), Polo-Like Kinase 1 and 4 (PLK1 and PLK4), Centrosomal Protein 152 (CEP 152) and Cyclin B1 (CCNB1) which had an increase in fold change of 1.69, 1.89, 1.61, 1.18 and 1.72 respectively, as identified by RNA-seq (Table 1). qRT-PCR validation of the target genes confirmed highly significant RNA-seq differential expression: ASPM, PLK1, PLK4, CEP152, and Cyclin B1 had an increase in fold change of 5.29, 10.65, 10.97, 3.76, and 20.16 respectively (Figure 3 Top). In the BTCOE-4795 cell line, qRT-PCR identified increased expression of the same targets, i.e. ASPM, PLK1, PLK4, CEP152, and Cyclin B1 by a fold of 1.18, 1.33, 1.23, 1.17, and 4.56 respectively (Figure 3 Bottom). Importantly, the increased expression these genes (ASPM, PLK1, PLK4, CEP152, and Cyclin B1) due to GBMF was blocked in the presence of the T-type VGCC blocker ethosuximide (Supplementary Figure 6).

**Table 1:** Mitotic roles of Polo-Like Kinase - ingenuity pathway analysis

Mitotic roles of Polo-Like Kinase	
Gene	Fold change
Abnormal Spindle Microtubule Assembly (ASPM)	1.68 (Increase)
Polo-Like Kinase 1 (PLK-1)	1.89 (Increase)
Polo-Like Kinase 4 (PLK-4)	1.61 (Increase)
Centrosomal Protein 152 (CEP152)	1.18 (Increase)
CyclinBI (CCNB1)	1.72 (Increase)

Differentially expressed genes identified by RNA-seq analysis of U-251 cells exposed to GBMF or RCF for three hours daily for seven days. Abnormal Spindle Microtubule Assemble (ASPM), Po-lo-Like Kinase 1 and 4 (PLK1 and PLK4), Centrosomal Protein 152 (CEP 152) and Cyclin B1 (CCNB1) had increases in fold-change of 1.68 (FDR adjusted *p*-value: 0.0022), 1.89 (FDR adjusted *p*-value: 0.0022), 1.61 (FDR adjusted *p*-value: 0.0022), 1.18 (FDR adjusted *p*-value: 0.0443) and 1.72 (FDR adjusted *p*-value: 0.0022), respectively.

**Figure 4:**
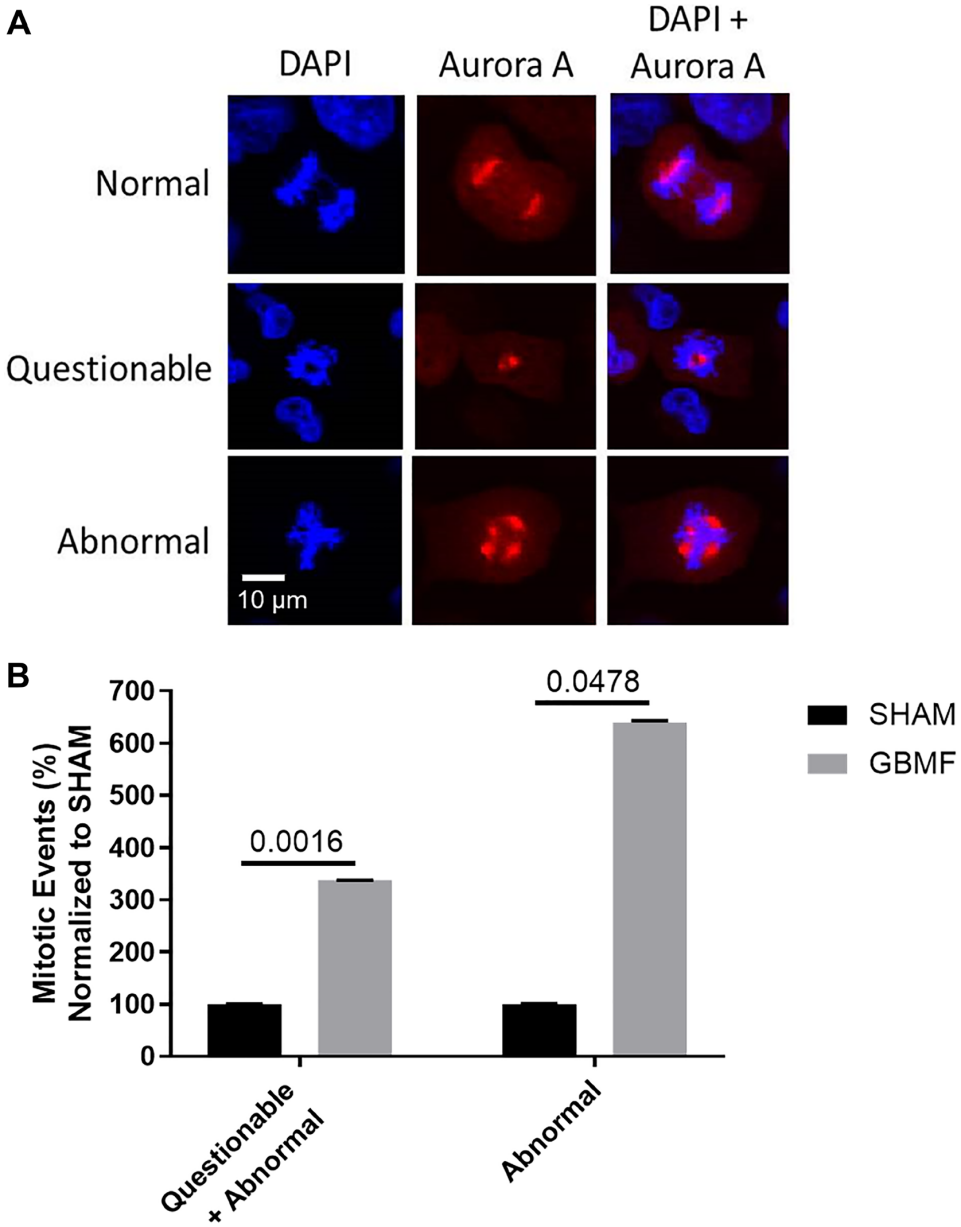
GBMF treatment disrupts the mitotic spindle(s) of GB cells. U251 cells were exposed to 27.12 MHz RF EMF amplitude modulated at GB specific frequencies three hours daily for seven days. (**A**) Mitotic spindle visualization and quantification by two blinded, independent investigators revealed that AM RF EMF treated cells exhibit a higher number of mitotic spindle disruption events than control cells. (**B**) There were significantly more mitotic events (Questionable+Abnormal) among the GBMF treated cells (366.67% increase) compared to the SHAM untreated cells; 2-tail *t*-test *p* = 0.0016. Additionally, when using the strictest analysis of mitotic events (Abnormal only) there was a significantly greater number of events among the GBMF treated cells (640% increase) compared to the SHAM untreated cells; 2-tail *t*-test *p* = 0.0478. Representative images/data shown.

Given the previously observed disruption of the mitotic spindle in hepatocellular carcinoma cells upon exposure to HCCMF [8] and the impact of ASPM, PLK1 and PLK4 on mitotic spindle formation, we hypothesized that GBMF might disrupt the mitotic spindle of GB cells. Mitotic spindles were identified as either proceeding normally, questionably or abnormally in U251 cells and were assessed blindly by two investigators (Table 2). As shown in Figure 4, GBMF exposure resulted in disruption of the mitotic spindle of U251 cells.

**Table 2:** GBMF treatment disrupts mitotic spindles of U251 cells

**U251 SHAM (*N* = 14 pictures)**		
Normal (*N* = 39)	(Q)uestionable (*N* = 4)	(A)bnormal (*N* = 39)
**U251 GBMF (*N* = 12 pictures)**		
Normal (*N* = 21)	(Q)uestionable (*N* = 11)	(A)bnormal (*N* = 4)
	**2-tail *t*-test**	***p*-value **
	**SHAM (Q+A) vs. GBMF (Q+A)** **SHAM (Abnormal) vs. GBMF (Abnormal)**	**0.0016** **0.0478**

U251 SHAM pictures *N* = 14. In the 14 pictures there were *N* = 39 – ‘Normal’, *N* = 4 – ‘Questionable’, and *N* = 1 – ‘Abnormal’ mitotic events identified. U251 GBMF pictures *N* = 12. In the 12 pictures there were *N* = 21 – ‘Normal’, *N* = 11 – ‘Questionable’, and *N* = 4 – ‘Abnormal’ mitotic events identified. Representative experiment shown. Experiment repeated twice.

### Clinical response to GBMF

**Figure 5:**
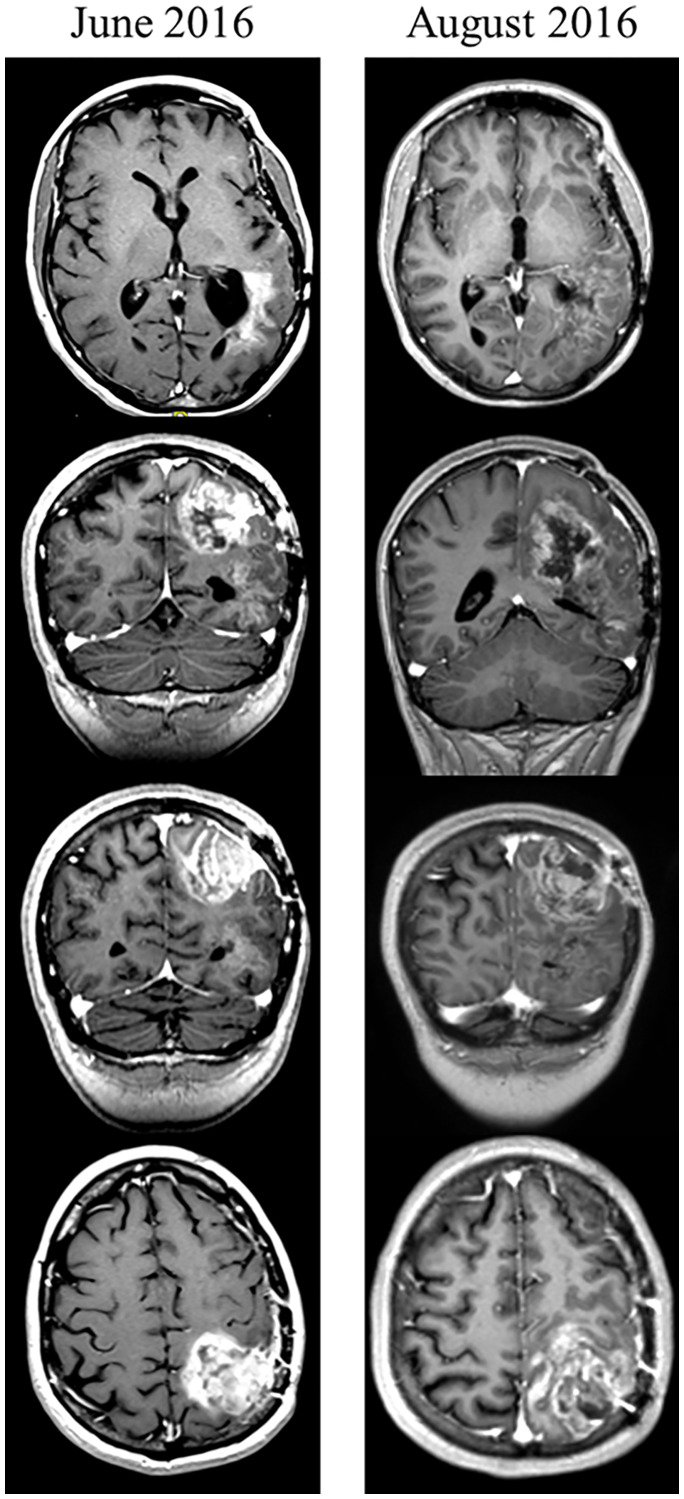
Clinical response to GBMF – representative images. 38-year-old woman diagnosed with gliosarcoma with components of GB (*IDH1* and *IDH2* wt, EGFRvIII negative recurrent GB) in April 2014. She underwent resection of the tumor and received treatment with radiation therapy and temozolomide. She was subsequently treated with nivolumab, then bevacizumab and fotemustine. She was also on a ketogenic diet. Patient had progression of disease prior to initiation of treatment with GB-specific AM RF EMF. Axial and coronal T1 post-contrast images in March 2016 revealed a left temporal enhancing mass which demonstrated progression on follow-up images obtained in June, 2016 at which time the patient began compassionate use of the treatment device (TheraBionic). Subsequent imaging in August 2016 demonstrated a less solid enhancement pattern (heterogeneous pattern/ill defined) which was felt to represent a treatment effect and not tumor progression. Treatment was stopped after 3 months due to intracranial bleeding thought to be unrelated to GBMF treatment. Patient expired November 2016. Data was interpreted by Annette Johnson MD, Department of Radiology. The patient received compassionate treatment (beginning July 4th, 2016) with the TheraBionic^®^ device emitting GBMF three hours daily and had follow up imaging August 18th, 2016.

The patient was a 38-year-old woman diagnosed with gliosarcoma with components of GB (*IDH1* and *IDH2* wt, EGFRvIII negative recurrent GB) in April 2014. She underwent resection of the tumor and received treatment with radiation therapy and temozolomide. She was subsequently treated with nivolumab, then bevacizumab and fotemustine. She was also on a ketogenic diet. The patient had progression of disease prior to initiation of treatment with GB-specific AM RF EMF (Supplementary Figure 7). Treatment with the TheraBionic^®^ device programmed to administer GBMF was initiated on July 4, 2016. At the follow up visit on August 5 the patient’s spouse reported improvement in word recognition. He also reported that the patient was able to dance at a party, a significant functional improvement. However, she complained of persistent left upper and lower extremity weakness. As shown in Figure 5 the scan taken on the 18th of August 2016 demonstrates a decreased intensity and amount of enhancement as compared with the initial scan taken the 20th of June 2016. The left temporal enhancing mass demonstrates a much less solid enhancement pattern over a somewhat larger area and is difficult to measure. The left parietal enhancing mass has increased in size but shows a much more heterogeneous pattern of more ill-defined enhancement. The findings are suggestive of treatment effect rather than tumor progression Figure 5. Treatment was stopped after three months because of intracranial bleeding felt to be unrelated to the AM RF EMF treatment. The patient expired November 22nd, 2016.

A second patient was a 47-year-old male incidentally diagnosed with a left parietal brain tumor at the age of 32 following a fall. Following two years of monitoring, he underwent a left parietal craniotomy and subtotal resection of the lesion in 2007 which confirmed the presence of a grade II oligodendroglioma with 1p and 19q deletions. He next received 12 months of temozolomide with subsequent progression by MR imaging prompting a second surgery which confirmed the original diagnosis. He then received concurrent radiation and temozolomide followed by one cycle of adjuvant monthly temozolomide which was discontinued by patient preference (Supplementary Figure 8).

Following three years of surveillance, he experienced three further progressions of his tumor treated with metronomic temozolomide, carboplatin, irinotecan and bevacizumab, and then maintenance bevacizumab following VP shunt placement for obstructive hydrocephalus in 2019. In light of further clinical and radiographic deterioration, a request for compassionate use of the TheraBionic device was asked for and granted by the FDA on June 5th, 2020. The patient and his mother were educated on how to use this therapy and he began treatment with the TheraBionic device on July 1st, 2020. He continued on the combination of the TheraBionic device used three times daily and bevacizumab given every 2–3 weeks until late in October of 2020 when the device was discontinued in the setting of clinical deterioration. MR imaging after two months (Supplementary Figure 8) of the TheraBionic use showed stable disease and the patient tolerated the treatment well. He had no adverse events while on TheraBionic other than some transient mouth discomfort early in the treatment course treated effectively with several days of chlorhexidine mouthwash. Note: this patient was receiving bevacizumab at the time of MR imaging which reduces enhancement thereby impacting the image.

## DISCUSSION

This is the first report showing that 27.12 MHz RF EMF, which are amplitude-modulated at GB-specific frequencies (GBMF) identified in patients with primary brain tumors [5], have antiproliferative effects in several GB cell lines. The antiproliferative effect was observed in patient-derived cell lines (BTCOE -4765 and -4795) in addition to the U251 cell line. At this time, it is unknown to the authors why the cell line, BTCOE-4536, was unaffected by GBMF treatment and we can provide no reasonable rationale. The magnitude of GBMF antiproliferative effect (15–34%) is comparable to that observed with hepatocellular carcinoma-specific frequencies in five hepatocellular carcinoma cell lines (19–47%) [7, 8] and with breast cancer-specific frequencies in six breast cancer cell lines (10–20%) [8, 9], respectively. GBMF cancer stem cell inhibitory effects (36–56%) are also in the same range as observed in hepatocellular carcinoma and breast cancer cell lines after exposure to tumor specific frequencies, i.e., 38–58% and 27–79%, respectively. The experiments demonstrate that only GBMF-specific frequencies have antiproliferative and cancer stem cell inhibitory effects. Indeed, treatment with hepatocellular carcinoma-specific frequencies (HCCMF) was indistinguishable from SHAM exposure.

An agnostic genomic approach led to the discovery that GBMF block the growth of GB cells by modulation of the “Mitotic Roles of Polo-Like Kinase” pathway. This pathway is involved in regulating cell cycle kinetics and has been identified as a potential therapeutic target in GB. Modulation of the “Mitotic Roles of Polo-Like Kinase” pathway is linked to disruption of the mitotic spindle of GB cells [20, 21]. To further characterize GBMF impact on this pathway, we investigated the biology of centrioles. CEP-152 and PLK-4 are essential for the genesis of centrioles where CEP-152 recruitment leads to PLK-4 scaffold switching and the repositioning of PLK-4, leading to the completion of the daughter centriole in the G1 phase of mitosis. The increase in mitotic spindle disruption, the overexpression of CEP152 and PLK-4, or potentially the suppression of the degradation of PLK-4, can lead to an increase in the number of centrioles and hence each mother centriole is able to nucleate more than one daughter centriole at a time leading to disruption of the mitotic mechanism [20, 22]. Moreover, published data has shown that modulation to PLK-4 expression can cause distress in mitotic fidelity [23–26].

**Figure 6:**
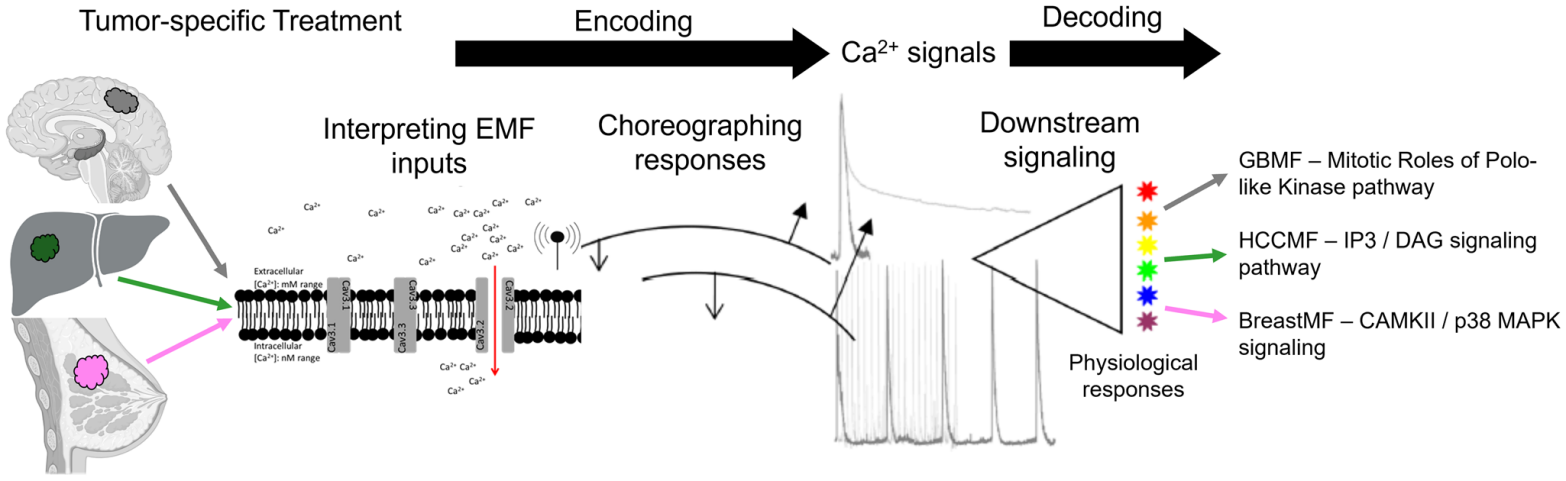
Tumor-specific AM RF EMF modulation/demodulation. Tumor-specific frequency treatment (i.e. a modulated anti-cancer signal) induces Ca^2+^ entry (interpreting EMF inputs) into a cancer cell (i.e., glioblastoma, hepatocellular carcinoma, or breast cancer). This treatment event encodes and choreographs the anti-cancer response that we see *in vitro, in vivo,* and clinically. Tumor-specific frequencies take the place of physical cues i.e. the encoding portion of a receptor, such as when a ligand binds to its corresponding receptor, and Ca^2+^ influx/signaling occurs. The end result is a decoding (demodulation) of the Ca^2+^ signaling with a series of composed responses (downstream signaling) which end by inducing physiological response/biological effects (i.e. activation of specific signaling pathways). Image created with https://www.biorender.com/.

This report demonstrates that GBMF antiproliferative and cancer stem cell inhibitory effects are contingent upon Ca^2+^ influx through CACNA1H, the Ca_v_3.2 T-type VGCC. While CACNA1H is the same bioantenna for tumor-specific AM RF EMF identified in patients with tumors arising from the breast, liver, and brain [5, 7, 9], Ca^2+^ influx through CACNA1H into cancer cells result in the activation of different signaling pathways in these three tumor types. Breast cancer-specific frequencies activate CAMKII/p38 MAPK in breast cancer [9], hepatocellular carcinoma-specific frequencies activate IP3/DAG in hepatocellular carcinoma [7], and GB-specific frequencies activate Mitotic Roles of Polo-Like Kinase in GB as shown in this report. The tumor-specific frequency sets for HCC, breast cancer, and GB are more than 60% different in their makeup [5]. Tumor-specific frequencies appear to be a necessary component of the antiproliferative effects observed in hepatocellular carcinoma [7, 8], breast cancer [8, 9], and in GB as shown in this report. Indeed, no antiproliferative effect was observed when tumor cells were exposed to non-corresponding tumor-specific frequencies. We therefore postulate that tumor-specific frequencies trigger a specific set of instructions that only the matching ‘frequency set - cancer type’ can accurately interpret (Figure 6). Demodulation of tumor-specific frequencies is likely to result in specific patterns of Ca^2+^ influx. If tumor-specific frequency sets are a unique signature and Ca_v_3.2 the point of demodulation of the signal, then does the tumor-specific frequency signature translate into a specific pattern and intensity of Ca^2+^ puffs? This is highly possible as Ca^2+^ signaling kinetics impact how information is processed to encode/decode Ca^2+^ signals, the choreography of responses to ensure specific and efficient signaling, and the overall temporal gearing so that Ca^2+^ signals have lasting physiological effects [27]. It has been previously described that gene transcription of B lymphocytes is achieved through amplitude modulation of calcium signaling [28, 29]. Moreover, Ca^2+^ oscillations can evoke enhanced gene expression compared to a ‘fixed level’ in lymphocytes [30]. Dolmetsch et al. demonstrated how downstream effectors can decode information contained in the amplitude and duration of calcium signals [29]. More broadly, Ca^2+^ has the ability to transmit information through the use of frequency modulation and amplitude modulation [28]. Restricting the influx signaling through the Ca_v_3.2 channel would allow for delivery of information that displays spatiotemporal malleability and is consistent with a discrete and specific response. Hence, we propose cancer cells respond to tumor-specific frequencies by decoding the modulation frequencies into Ca^2+^ signals, which block tumorigenic pathways within the cancer cell (Figure 6).

While different frequencies activate different pathways in specific tumor types, the nature and magnitude of the resulting anticancer effects observed are similar, i.e., reduced stemness, lack of apoptosis, proliferative inhibition, mitotic spindle disruption, and tumor shrinkage or tumor stabilization in patients [7–9]. This suggests that tumor-specific frequencies result in similar end effects in various tumor types through activation of tumor-specific pathways.

Additionally, tumor-specific AM RF EMF are identified in patients with similar tumor type regardless of subtype, e.g., breast cancer-specific frequencies include frequencies from breast cancer patients of various subtypes (ER+/PR+, Triple Negative, Claudin low, etc.) [5]. Hence, glioblastoma-specific frequencies could be used to treat all forms of glioblastoma. Glioblastoma-specific frequencies would not, however, be used to treat meningiomas. To treat meningiomas, meningioma-specific frequencies would have to be identified from patients with a diagnosis of meningioma [5]. Moreover, tumor-specific frequencies do not have an impact on non-malignant tissues, so normal healthy cells would go unaffected [7–9].

Similarly, to what has been observed in patients with breast cancer and hepatocellular carcinoma, this report shows feasibility of this treatment approach in patients with malignant glioma and provides evidence of anticancer activity in one of them. We have previously reported a dosimetry analysis of the systemic delivery of intrabuccally administered AM RF EMF. We characterized the overall specific absorption rate (SAR i.e. the measure of the rate of energy absorbed by the body when exposed to RF EMF) as well as the organ-specific SAR [7]. Briefly, the whole body SAR (wbSAR) of AM RF EMF is 1.35 mW/kg with a peak spatial SAR (psSAR), over 1 g of tissue, ranging from 146 to 352 mW/kg [7]. These values are well under the International Commission on Non-Ionizing Radiation Protection (ICNIRP) standard safety limits (whole body SAR of 80 mW/kg or peak spatial SAR of 2000 mW/kg) [31]. In assessing the organ-specific SAR values we found that brain gray matter (0.04–0.20 mW/kg), brain white matter (0.02–0.0.10 mW/kg), and the midbrain (0.06–0.20 mW/kg) have mean SAR ranges that again fall well under the ICNIRP standard safety limits [7]. The clinical data reported here provide additional evidence that AM RF EMF is a targeted systemic therapy as shown by its ability to block tumor growth in the brain [9], bone [5], adrenal gland [5], liver [5, 7, 16], and lung [16].

Tumor Treating Fields (TTF) has been a paradigm change in oncology, especially as it relates to the treatment of glioblastoma [3, 14] and, more recently for the treatment of mesothelioma [32]. TTF (Novocure device) and AM RF EMF (TheraBionic device) are both categorized by Medicare as Durable Medical Equipment for the treatment of cancer. While TTF and AM RF EMF are fundamentally different as it relates to the signal delivered to the human body, there are some similarities with respect to their respective mechanism of action such as mitotic spindle disruption [10]. Intratumoral Ca^2+^ influx has been reported upon exposure of GB cells to TTF [15]. However, Ca^2+^ influx depends on CACNA1C with TTF [15] while it depends on CACNA1H with AM RF EMF as shown in this report.

In summary, while the two reported cases have limitations and only represent preliminary data, they demonstrate the feasibility of this novel treatment approach in patients with primary brain tumors. AM RF EMF antitumor activity in several GB cell lines warrant preclinical as well as clinical studies of the TheraBionic device in this patient population [7, 9].

## MATERIALS AND METHODS

### AM RF EMF exposure *in vitro*

Treatment of patients with intrabuccally-administered AM RF EMF results in whole body mean Specific Absorption Rate (SAR) ranging from 0.2 to 1 mW/kg, with peak spatial SAR over 1 g ranging from 150 to 350 mW/kg [7], which are well below international guidelines for safety exposure [33]. Cell lines were exposed to 27.12 MHz radiofrequency electromagnetic fields using exposure systems replicating *in vivo* exposure levels [8, 34]. EMF treatment is non-thermal and non-ionizing. Experiments were conducted at an SAR of 30 and 400 mW/kg to replicate the SAR in humans. Cells were exposed for three hours daily, seven days in a row. Cells were exposed either to tumor-specific modulation frequencies that were previously identified in patients with a diagnosis of GB (GBMF) or were not exposed to any EMF (SHAM). As control for tumor-specific frequencies, cells were exposed to either hepatocellular carcinoma-specific frequencies (HCCMF) or randomly chosen frequencies (RCF) as described previously [7–9].

### Cell lines

The Debinski Laboratory/Brain Tumor Center of Excellence (BTCOE) at Wake Forest University School of Medicine provided all cell lines: BTCOE-4765 (Female), BTCOE-4536 (Female), BTCOE-4795 (Male), and U251. All (BTCOE) cells were grown in RPMI-1640 media containing 10% HI-FBS and glucose adjusted to 4.5 g/L. U251 cells were grown in DMEM media containing 10% HI-FBS and 5 mL NEAA. The established BTCOE cell lines were genetically identical to the BTCOE tumor samples of origin. All cell lines were maintained under standard conditions. The BTCOE-4765 cell line has the following short tandem repeat profile: AMEL (X); CSF1PO (11,12); D13S317 (13); D16S539 (12); D18S51 (14,16); D21S11 (30, 31.2); D3S1358 (15, 18); D5S818 (12,13); D7S820 (12,13); D8S1179 (12); FGA (22,24); Penta_D (11,14); Penta_E (7); TH01 (8,9); TPOX (8, 10); vWA (19). The BTCOE-4536 cell line has the following short tandem repeat profile: AMEL (X); CSF1PO (12); D13S317 (9, 11); D16S539 (11, 13); D18S51 (13, 15); D21S11 (29); D3S1358 (15, 17); D5S818 (10, 13); D7S820 (12); D8S1179 (8, 13); FGA (22); Penta_D (12, 15); Penta_E (18, 22); TH01 (7, 9.3); TPOX (8, 11); vWA (17, 18). The BTCOE-4795 cell line has the following short tandem repeat profile: AMEL (X, Y); CSF1PO (11, 12); D13S317 (13); D16S539 (12, 14); D18S51 (13, 15); D21S11 (28, 30.2); D3S1358 (15, 16); D5S818 (12); D7S820 (9, 11); D8S1179 (12); FGA (22); Penta_D (9, 13); Penta_E (5); TH01 (9.3); TPOX (8, 11); vWA (17, 18). The U251 cell line has the following short tandem repeat profile: AMEL (X, Y); CSF1PO (11, 12, 13); D13S317 (10,11); D16S539 (12); D18S51 (13); D21S11 (29); D3S1358 (16,17); D5S818 (11,12); D7S820 (10,12); D8S1179 (13,15); FGA (22,25); Penta_D (12); Penta_E (7,10); TH01 (9.3); TPOX (8); vWA (16,18).

### (3H) thymidine incorporation assay

Growth inhibition (GI) was assessed in cell lines after treatment with GB-specific modulation frequencies as previously described [7]. Briefly, following six days of AM RF EMF exposure for 3 hours daily, on the seventh and final day of exposure 3 μCi ^3^H Thymidine (Perkin-Elmer) is added to each well, i.e., ^3^H concentration = 1 μCi of ^3^H per mL of media, and then the final exposure session (3 h long @ 37 °C) takes place with one additional hour of incubation at 37 °C without AM RF EMF exposure. Following the 4 h of total incubation time, the ^3^H containing media is removed, and the 35 mm dishes or six-well plates are washed with cold PBS for 5 min with constant gentle rocking/agitation. After 5 min, PBS is removed and 800 uL of 0.2 N NaOH is added to each well/dish. Cells are placed on a rocker for a minimum of 1 h, up to overnight, with gentle agitation to lyse cells. Afterwards, lysate is transferred to a 7 mL scintillation vial containing 4 mL of Ultima Gold (Perkin Elmer) scintillation fluid and read with a scintillation counter (Beckman Coulter).

### Luminescent cell viability assay

Cell proliferation was quantitated using the Promega Cell Titer-Glo Luminescent Cell Viability Assay (Promega, Madison, WI, USA), a method to determine the number of viable cells in culture based on ATP quantitation.

### Western blots

U251 and BTCOE-4795 cells were seeded in six-well plates at 10,000 and 60,000 cells per dish, respectively, and cultured in the presence or absence of a pan T-type VGCC 2-ethyl-2- methyl succinimide (ethosuximide, ETHOS group) (1 mM) (Sigma-Aldrich). The treatment groups were as follows: SHAM (Vehicle), GBMF (Vehicle), SHAM (ETHOS), and GBMF (ETHOS). Cells were cultured and treated with GB-specific AM RF EMF for 7 days, followed by cell lysis (Thermo Scientific, Cat #89901) and protein quantification (Thermo Scientific, Cat #2352). Images were generated and captured by using the Thermo Scientific myECL Imager (model # 62236x) to process western blots. 15 μg of protein were used per lane. Apoptosis targets: ABCAM apoptosis cocktail (ABCAM; ab136812): Cleaved PARP - 89 kDa, Muscle Actin - 42 kDa, Procaspase 3 - 32 kDa, and Cleaved caspase 3 - 17 kDa. HeLa Apoptosis lysate set: Staurosporine-Treated and Vehicle-treated control (ABCAM; ab136806). All experiments were repeated at least twice.

### T-type VGCC blockade

U251 and BTCOE-4795 cells were seeded in six-well plates at 10,000 and 60,000 cells per dish, respectively, and cultured in the presence or absence of a pan T-type VGCC 2-ethyl-2- methyl succinimide (ethosuximide, ETHOS group) (1 mM) (Sigma-Aldrich). Ethosuximide (ETHOS) was dissolved in 100% ethanol (Fisher) as per Sigma-Aldrich recommendation to create working solution. 100% ethanol is the vehicle control (Vehicle). The treatment groups were as follows: SHAM (Vehicle), GBMF (Vehicle), SHAM (ETHOS), and GBMF (ETHOS). Cells were left to adhere overnight and were then cultured in their corresponding media. Cells were exposed to either GBMF daily for 3 h in a row or received no treatment, either in the presence or in the absence of ethosuximide (final concentration 1 mM). Ethosuximide working solution was added to the culture medium within 10 min before exposure to SHAM or GBMF groups. Within 5 min after completion of the three-hour exposure time, media was removed from all dishes and replaced with fresh media without ethosuximide. On day seven, cell proliferation was assessed with the tritiated thymidine incorporation assay, flow cytometry markers (Nestin and CD133) were stained, or cells were cultured in tumor sphere media for sphere formation assays.

### Sphere-forming assay

Cells were plated (200 cells/well) in 96-well ultra-low attachment plates (Corning) with DMEM/F12 supplemented with 2% B27 (Invitrogen), 20 ng/ml EGF (Sigma Aldrich), and 4 μg/ml insulin (Sigma-Aldrich). The number of spheres were counted by hand at day 7, and data were represented as the means ± SEM.

### Colony formation assay

U251 cells were plated (200 cells/well) in 6-well plates (Corning) and cultured in DMEM media containing 10% HI-FBS and 5 mL NEAA. Cells were then treated with GB-specific AM RF EMF for seven days in a row, three hours per day. On the 7th and final day, following GBMF treatment, cells were stained with crystal violet stain as follows. Media was removed from each well (no wash occurred) and Crystal violet stain was added to each well (~1 mL/well). 6-well plates were then placed in the dark for 20 minutes at room temperature. Crystal violet stain was then removed, and all wells were washed by gently adding distilled water (~1 mL/well) alongside the well wall and gently shaking (by hand) for 15 seconds (performed twice). 6- well plates were then placed on paper towels to air dry upside down (overnight). The following day colonies (>50 cells) were counted by hand. Data is represented as the means ± SEM. Components of Crystal Violet Cell Colony staining: 0.5 g Crystal Violet (0.05% w/v), 27 ml 37% Formaldehyde (1%), 100 ml 10X PBS (1x), 10 ml Methanol (1%), 863 ml dH_2_O to bring up solution to 1L.

### shRNA knockdown of T-type voltage-gated calcium channels

The specific knockdown of all three T-types VGCC isoforms in U251 was accomplished by using the following kits. CACNA1g Human shRNA Plasmid Kit (Locus ID 8913) (Cat# TL305680 ORIGENE); CACNA1h Human shRNA Plasmid KIT (Locus ID 8912) (Cat# TL314243 ORIGENE); CACNA1i Human shRNA Plasmid Kit (Locus ID 8911) (Cat# TL314242 ORIGENE).

### Confocal laser scanning microscopy of mitotic spindles

Cells undergoing mitosis were imaged using an Olympus FV1200 SPECTRAL Laser scanning confocal microscope with an Olympus IX83 inverted platform (Olympus, Tokyo, Japan). For imaging experiments, U251 cells were grown in sterile 35 mm optical glass bottom cell culture dishes (ibidi μ-Dish, Cat#81156, ibidi USA, Inc., Fitchburg, WI). U251 cells were initially plated at a concentration of 5,000 cells per mL in 3 mL of media. Once the cells were given 8–18 hours to attach to the cover glass, they were exposed to AM RF EMF exposure 3 hours a day for 7 consecutive days.

Following AM RF EMF exposure, indirect immunofluorescent microscopy was used to compare the cells receiving GB-specific modulation frequencies with cells not receiving any exposure (Aurora A/AIK (1G4) – used for mitotic spindle visualization) Rabbit mAb #4718, Cell Signaling Technology Inc., Danvers, MA; Goat anti-Rabbit IgG (H+L) Cross-Adsorbed Secondary Antibody, Alexa Fluor 594, A-11012, ThermoFisher Scientific, Waltham, MA; SlowFade^®^ Diamond Antifade Mountant with DAPI, Catalog# S36964, ThermoFisher Scientific, Waltham, MA, USA).

### Flow cytometry

Cells cultured, divided, and treated with SHAM or GBMF (in the presence or absence of ethosuximide 1 mM). After seven days of treatment cells were labelled for NESTIN-AF488 (mouse anti-human 1:50 (U251 and BTCOE-4795), Cat#53-9843 Affymetrix eBioscience) and CD133-APC (mouse anti-human 1:100 (U251 and BTCOE-4795) Cat# 130–098-826 Miltenyi Biotec) markers of cancer stem cell, fixed and analyzed via flow cytometry. Data collection was performed on a C6 accrui flow cytometer while analysis was performed on CFlow Plus software (Becton Dickinson).

### Gene expression analysis

RNA extraction from cells was performed using RNeasy Mini Kit (QIAGEN). qRT-PCR was performed using a Roche LightCycler II and 1-Step Brilliant II SYBR Green qRT-PCR master mix kit (Agilent Technologies). Samples were run at 30 ng mRNA, according to manufacturer protocol. Roche LightCycler software was used to calculate/analyze relative quantification of gene expression. We have used the Δ ΔCT method to calculate fold change. Melting curves reported by the Roche LightCycler software were used to verify fidelity of the PCR product. Relative gene expression (Fold Change) for qRT-PCR were expressed as mean ± SEM [35].

### RNA-sequencing

RNA-sequencing was performed by the Wake Forest Baptist CCC Cancer Genomics Shared Resource. RNA was purified from cells using the miRNA mini kit from Qiagen. RNA integrity number (RIN) was determined by electrophoretic tracing using an Agilent Bioanalyzer. RNA-seq libraries were constructed for samples (RIN >8.0) using the Illumina TruSeq Stranded Total RNA kit with Ribo-Zero rRNA depletion. Indexed libraries were sequenced using an Illumina NextSeq 500 DNA sequencer programmed for 150 × 150 nt paired end reads, generating >50 million reads per sample with >75% of sequences achieving >Q30 Phred quality score. This sequencing depth and quality are optimal for analysis of differentially expressed genes (DEGs) and allele-specific gene expression, and read lengths are sufficient to detect splice variations, gene fusions, and long noncoding RNAs (lncRNAs).

### RNA-seq data analysis and identification of GBMF-related pathways and signaling networks

Read alignment was performed using the STAR sequence aligner, and gene counts determined using featureCounts software. Differential gene expression was analyzed using DESeq2 software. Six replicates per experimental condition were performed, and all experimental conditions prepared in the same experiment, to have sufficient power to detect DEGs at each time point using DESeq2.25. Significant DEGs were conservatively defined as *p* < 0.05 after adjustment for false discovery. DEGs were analyzed for significant enrichment of biological pathways and signaling networks using Ingenuity Pathway Analysis (IPA), Causal Network Analysis and Upstream Regulator Tools, and DAVID software.

### PCR primers and machine protocol

ASPM-Forward primer: 5′ GAG AGA GAG AAA GCT GCA AGA A 3′
ASPM-Reverse primer: 5′ GAA TGA CGA GTG CTG CAT TAA C 3′
CEP 152-Forward primer: 5′ CAG CAG CTC TTT GAG GCT TAT 3′
CEP 152-Reverse primer: 5′ CAC AGC AGT CAC CTC CTT ATT C 3′
CYCLIN B1-Forward primer: 5′ GAT GCA GAA GAT GGA GCT GAT 3′
CYCLIN B1-Reverse primer: 5′ TCC CGA CCC AGT AGG TAT TT 3′
PLK 1-Forward primer: 5′ CAG CAA GTG GGT GGA CTA TT 3′
PLK 1-Reverse primer: 5′ GTA GAG GAT GAG GCG TGT TG 3′
PLK 4-Forward primer: 5′ TCA AGC ACT CTC CAA TCA TCT T 3′
PLK 4-Reverse primer: 5′ CAA ACC ACT GTT GTA CGG TTT C 3′
CACNA1g (CA_V_ 3.1)-Forward primer: 5′ CTT ACC AAC GCC CTA GAA ATC A 3′
CACNA1g (CA_V_ 3.1)-Reverse primer: 5′ GAT GTA GCC AAA GGG ACC ATA C 3′
CACNA1h (CA_V_ 3.2)-Forward primer: 5′ CAA GGA TGG ATG GGT GAA CA 3′
CACNA1h (CA_V_ 3.2)-Reverse primer: 5′ GAT GAG CAG GAA GGA GAT GAA G 3′
CACNA1i (CA_V_ 3.3)-Forward primer: 5′ GCC CTA CTA TGC CAC CTA TTG 3′
CACNA1i (CA_V_ 3.3)-Reverse primer: 5′ AGG CAG ATG ATG AAG GTG ATG 3′
GAPDH-Forward primer: 5′ TGC ACC ACC AAC TGC TTA GC 3′
GAPDH-Reverse primer: 5′ GGC ATG GAC TGT GGT CAT GAG 3′

Protocol: 1 cycle for 30 min at 50C, 1 cycle for 10 min at 95C, 40 × (30 sec at 95C/1 minute at 60C), Rest at 4C.

### Patient information

(1) 38-year-old woman with *IDH1* and *IDH2* wt, EGFRvIII- negative, recurrent GB s/p temozolomide, radiation therapy, ketogenic diet, nivolumab, bevacizumab, and fotemustine. The patient had surgery on October 1st, 2014 (left temporal lesion – gliosarcoma) and again on November 18th, 2015 (left parietal recurrence). The patient displayed progression of disease prior to initiation of treatment with GB-specific AM RF EMF and was not receiving any other treatment at the same time. The previous treatment (nivolumab) was discontinued four weeks before GB-specific AM RF EMF. Baseline imaging was obtained in March 2016 and progression of disease was noted in June 2016. Patient began compassionate treatment July 4th, 2016. (2) 47-year-old male patient with *IDH1* R132H mutated grade II oligodendroglioma with 1p and 19q deletions, s/p temozolomide, radiation therapy, carboplatin, irinotecan, and bevacizumab. No baseline images were obtained. Patient began compassionate treatment on July 1st, 2020.

### Statistical analysis

2-tail *t*-test was used to statistically compare the effects of experimental (GBMF) and control group (SHAM). Mann-Whitney test was used for the comparison of SHAM+Ethos and GBMF+Ethos in Figure 2E as the data for that comparison did not pass the assumptions of Normality (Shaprio-Wilk test *p*-value = 0.0027) and homogeneity of variances (Levene’s test *p*-value = 0.0331). One-way ANOVA was used to statistically compare the30ffectt of experimental groups (GBMF and HCCMF) and control group (SHAM). Post hoc testing was by the Tukey test. One-tail *t*-test was used to statistically compare the effect of experimental and control groups in qRT-PCR of the BTCOE-4795 cell line to validate the differentially expressed genes identified in the U251 cell line. Data are expressed as mean ± SEM. Graphpad Prism (version 6.0 and 10.0) was the software used for statistical analysis.

All experiments performed and reported were completed in accordance with relevant guidelines and regulations.

## SUPPLEMENTARY MATERIALS



## Abbreviations

^3^H: tritiated thymidine
AM RF EMF: Amplitude-Modulated Radiofrequency Electromagnetic fields
BTCOE: Brain Tumor Center of Excellence at Wake Forest Baptist
CACNA1C: Ca_v_ 1.2 L-type voltage-gated calcium channel
CACNA1H: Ca_v_3.2 T-type voltage gated calcium channel
DOI: Digital object identifier
FDA: United Stated Food and Drug Administration
g: gram
GB: Glioblastoma
GBMF: GB-specific Amplitude-Modulated Radiofrequency Electromagnetic fields
GI: Growth Inhibition
IPA: Ingenuity pathway analysis
HCCMF: hepatocellular carcinoma-specific frequencies
ICNIRP: International Commission on Non-Ionizing Radiation Protection
kHz: kilohertz
m: meter
MHz: Megahertz
mL: milliliter
mm: millimeter
MRI: Magnetic resonance imaging
mW/kg: milliwatt per kilogram
osSAR: organ-specific SAR
psSAR: peak spatial SAR
RCF: Randomly chosen frequencies (i.e. not tumor-specific)
RIN: RNA integrity number
SEM: Standard error of the mean
SAR: Specific absorption rate
SHAM: No EMF exposure group
TTFields: Tumor Treating Fields
μCi: microcurie
VGCC: Voltage gated Calcium Channel
wbSAR: Whole body SAR
wt: Wild type
