# Supplementary material for: Treatment of glioblastoma with tumor-specific amplitude-modulated radiofrequency electromagnetic fields
Source: Oncotarget. 2025 Oct 13;16:741–57. doi: 10.18632/oncotarget.28770 (PMC12517218; doi:10.18632/oncotarget.28770)
Supplement: Supplementary file 1 [file oncotarget-16-28770-s001.pdf]

Treatment of glioblastoma with tumor-specific amplitude-modulated radiofrequency electromagnetic fields

SUPPLEMENTARY MATERIALS

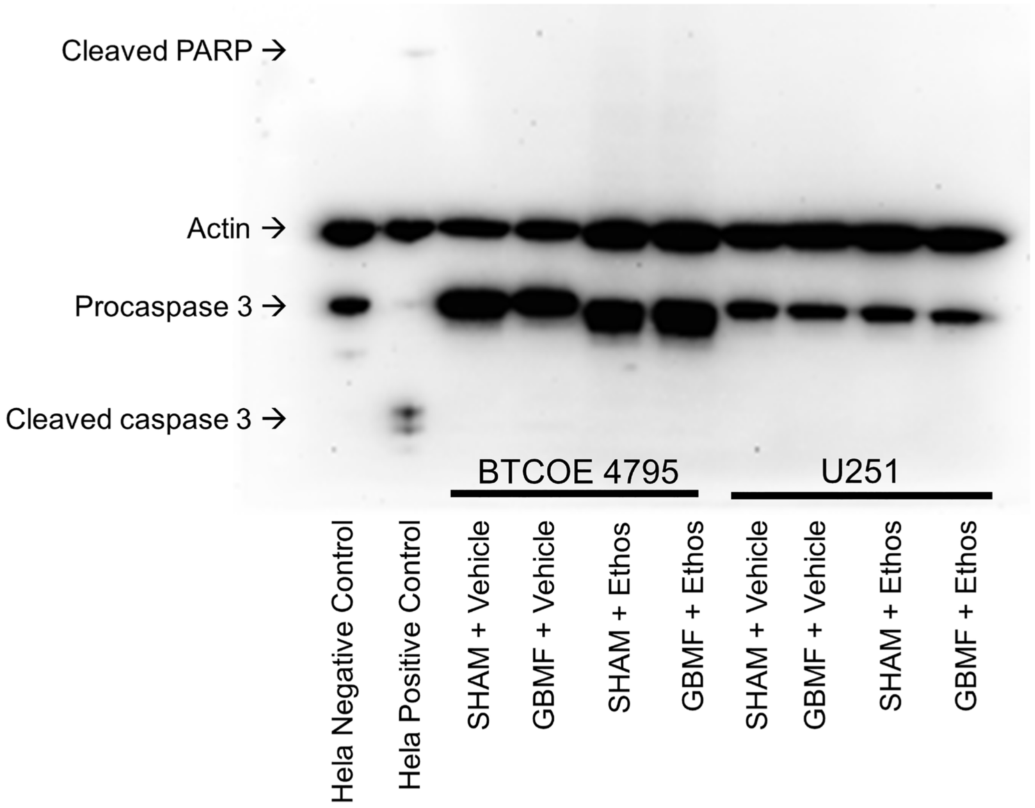

Supplementary Figure 1: Apoptotic activity of the BTCOE 4795 and U251 cell lines. Representative western blot image of apoptosis targets (Cleaved PARP - 89 kDa, Procaspase 3 - 32 kDa, Cleaved caspase 3 - 17 kDa, and Muscle actin - 42 kDa control). The BTCOE-4795 and U251 cell lines show no apoptotic activity in any of the exposure combinations (i.e., SHAM+Vehicle, GBMF+Vehicle, SHAM+Ethos, GBMF+Ethos) when compared to the controls. Image displayed was color inverted for clarity. Representative experiment shown. Experiments repeated twice.

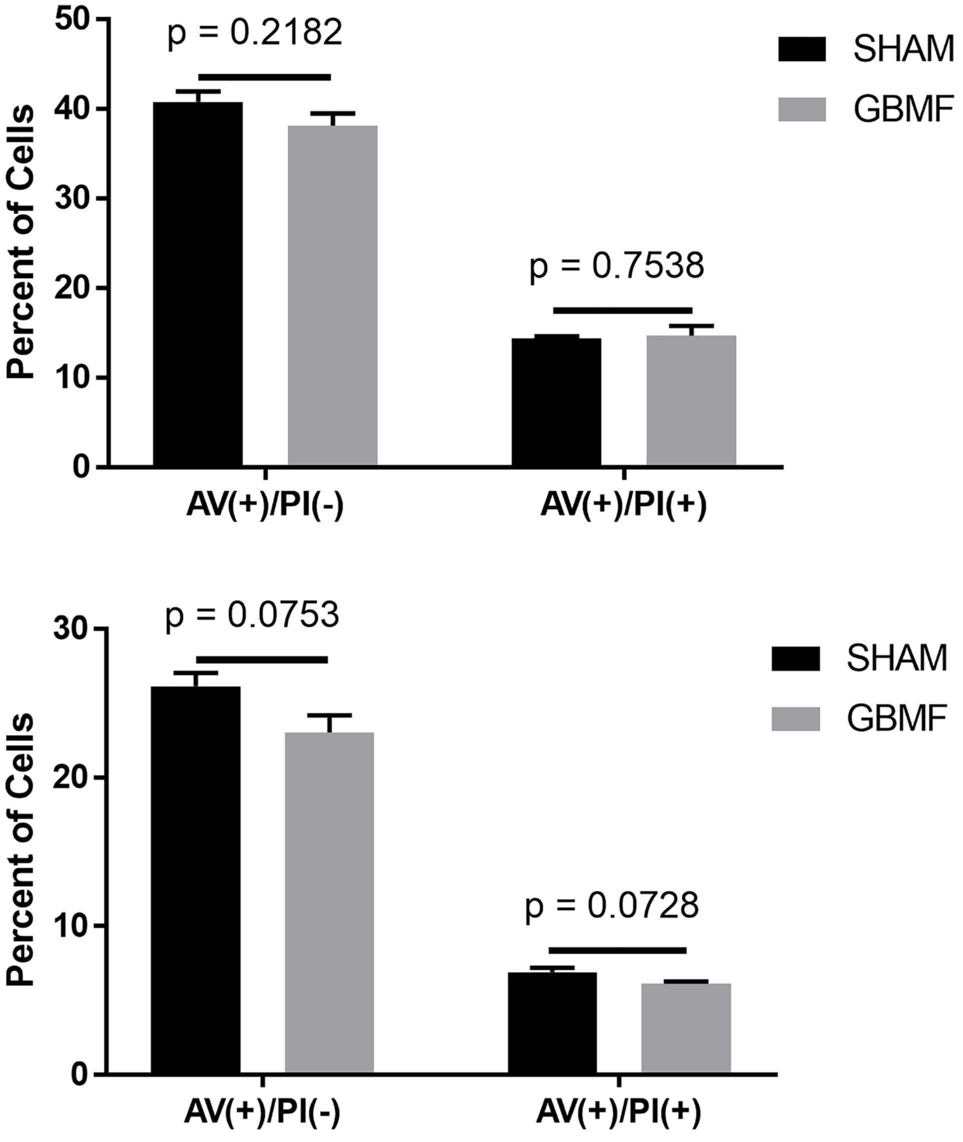

Supplementary Figure 2: Annexin V apoptotic detection of U251 and BTCOE-4795 cells. U251 Cell line (TOP Figure): AV(+)/PI(−) – Early Stage Apoptosis and AV(+)/PI(+) – End Stage Apoptosis show no statistical difference following GBMF exposure. BTCOE-4795 (BOTTOM Figure): AV(+)/PI(−) – Early Stage Apoptosis and AV(+)/PI(+) – End Stage Apoptosis show no statistical difference following GBMF exposure. Representative experiments shown. Experiments repeated twice.

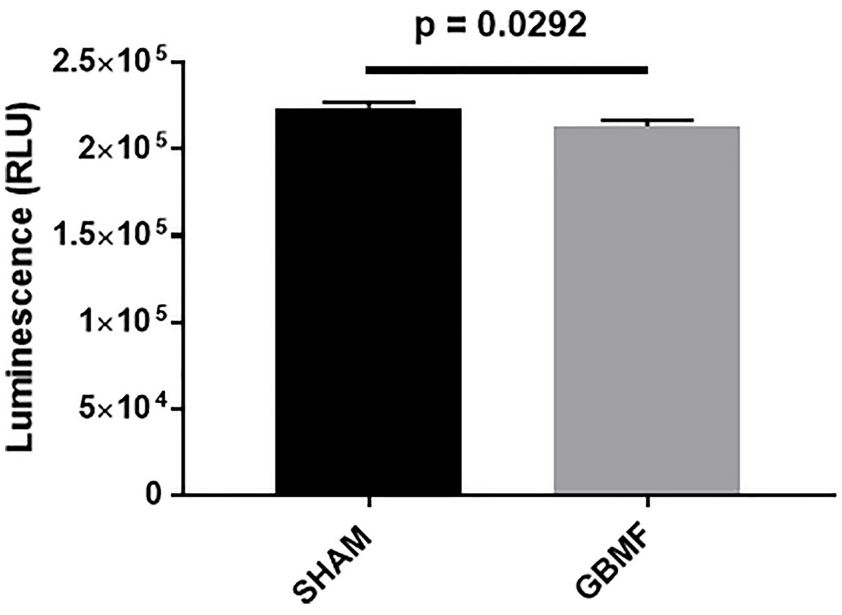

Supplementary Figure 3: Luminescent cell viability assay of the BTCOE-4795 cell line. 4.74% growth inhibition of BTCOE-4795 cells exposed to either SHAM or GB-specific frequencies (GBMF) for three hours daily for seven days. Two-tailed *t*-test *p*-value: 0.0292. Representative experimental data shown. Experiment performed twice (SHAM $N = 11$ and GBMF $N = 12$).

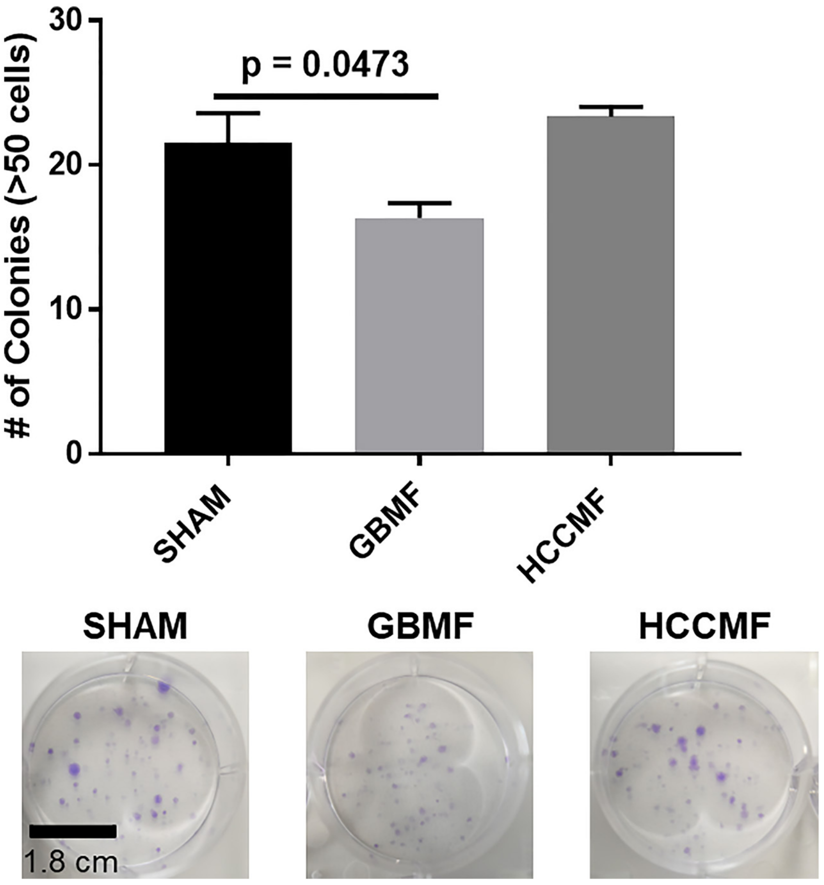

Supplementary Figure 4: Colony formation assay of the U251 cell line following treatment with SHAM, GBMF, and HCCMF. 24.03% fewer colonies in the GBMF treated group $N = 6$ per group. ANOVA ($F (2, 15) = 6.809$, $p = 0.0079$). Post hoc Tukey test: SHAM vs. GBMF $p = 0.0473$, SHAM vs. HCCMF $p = 0.6292$, GBMF vs. HCCMF $p = 0.0076$. Representative experiment shown. Experiments repeated twice.

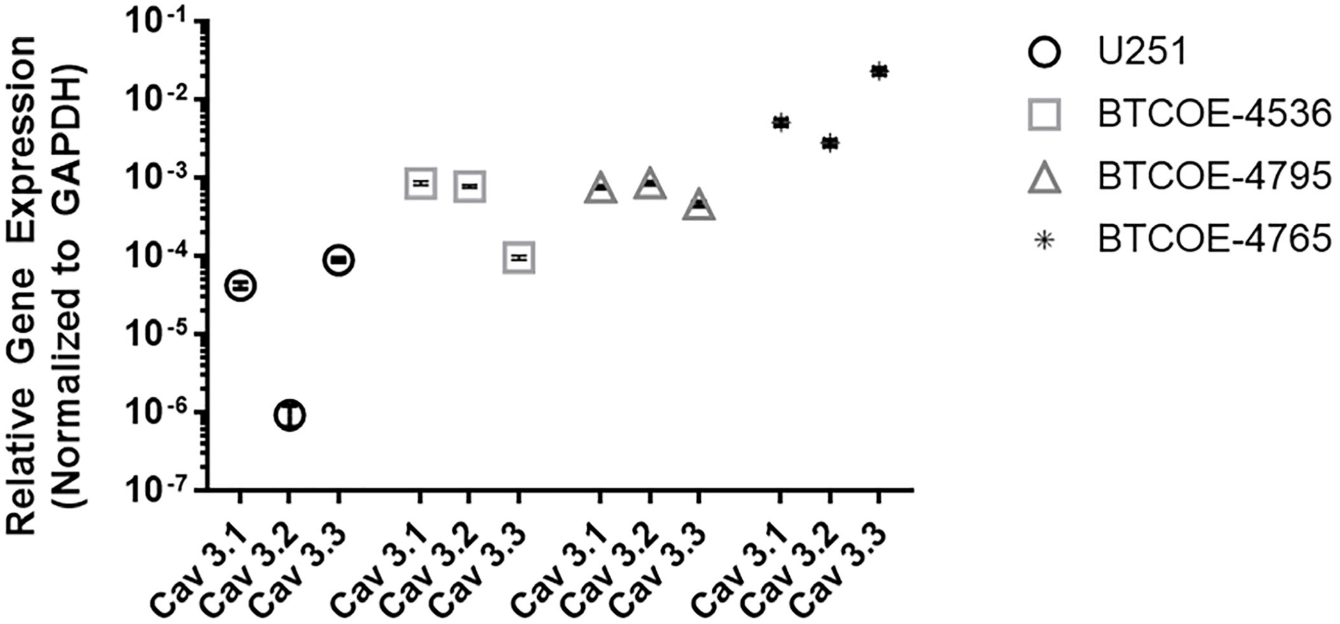

Supplementary Figure 5: Basal expression of T-type Voltage-Gated calcium channels in GB cell lines. qRT-PCR of U251, BTCOE-4536, BTCOE4795 BTCOE-4765.

U251:
Cav 3.1 – Mean: 4.14e-5, SEM: 3.84e-6, *N* = 12.
Cav 3.2 – Mean: 9.16e-7, SEM: 2.74e-7, *N* = 12.
Cav 3.3 – Mean: 8.77e-5, SEM: 5.42e-6, *N* = 12.
BTCOE-4536:
Cav 3.1 – Mean: 8.41e-4, SEM: 5.46e-5, *N* = 12.
Cav 3.2 – Mean: 7.61e-4, SEM: 3.47e-5, *N* = 12.
Cav 3.3 – Mean: 9.37e-5, SEM: 5.85e-6, *N* = 12.
BTCOE-4795:
Cav 3.1 – Mean: 7.46e-4, SEM: 2.84e-5, *N* = 12.
Cav 3.2 – Mean: 8.44e-4, SEM: 3.33e-5, *N* = 12.
Cav 3.2 – Mean: 4.55e-4, SEM: 3.08e-5, *N* = 12.
BTCOE-4765:
Cav 3.1 – Mean: 5.04e-3, SEM: 4.77e-4, *N* = 6.
Cav 3.2 – Mean: 2.76e-3, SEM: 2.63e-4, *N* = 6.
Cav 3.3 – Mean: 2.28e-2, SEM: 2.19e-3, *N* = 6.

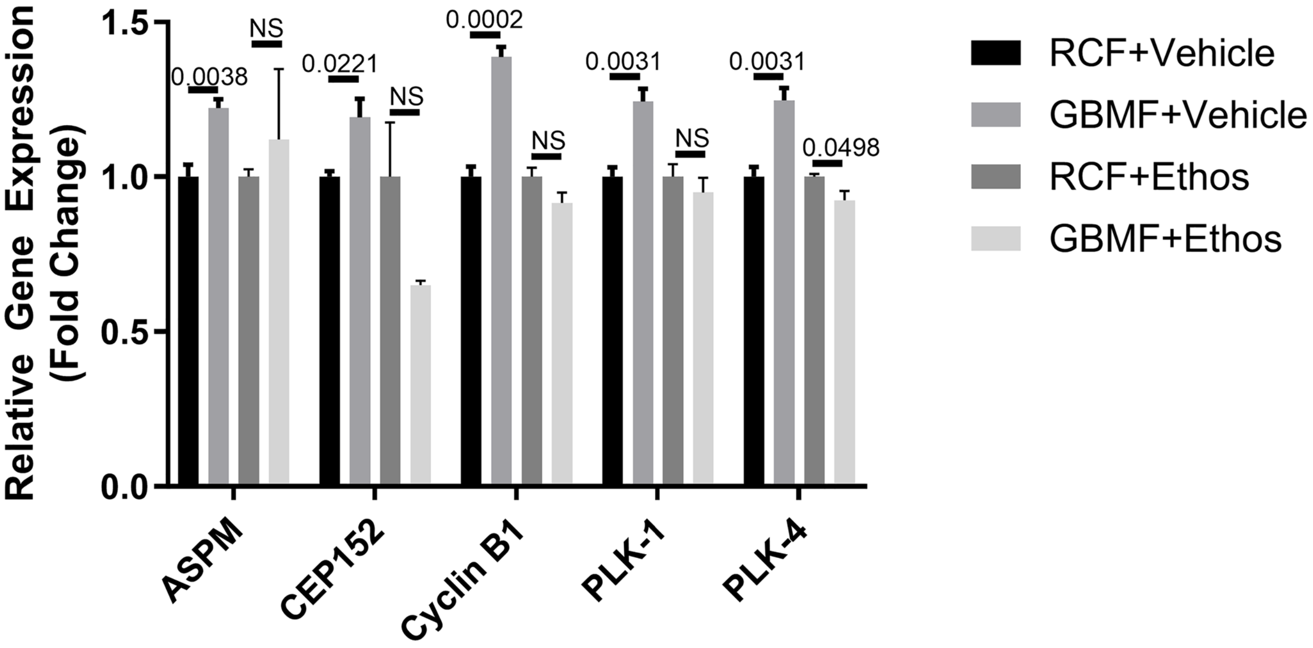

Supplementary Figure 6: qRT-PCR of five differentially-expressed genes in the presence or absence of ethosuximide. The increased expression of 5 genes following GBMF was blocked when U251 cells were cultured in the presence of ethosuximide. U251: ASPM – RCF+Vehicle ($N = 4$) vs. GBMF+Vehicle ($N = 4$) 2-tail T-test $p = 0.0038$. RCF+Ethos ($N = 4$) vs. GBMF+Ethos ($N = 4$) 2-tail T-test $p = 0.6222$. CEP152– RCF+Vehicle ($N = 4$) vs. GBMF+Vehicle ($N = 4$) 2-tail T-test $p = 0.0221$. RCF+Ethos ($N = 4$) vs. GBMF+Ethos ($N = 3$) 2-tail T-test $p = 0.1229$. Cyclin B1 – RCF+Vehicle ($N = 4$) vs. GBMF+Vehicle ($N = 4$) 2-tail T-test $p = 0.0002$. RCF+Ethos ($N = 4$) vs. GBMF+Ethos ($N = 4$) 2-tail T-test $p = 0.1034$. PLK1– RCF+Vehicle ($N = 4$) vs. GBMF+Vehicle ($N = 4$) 2-tail T-test $p = 0.0031$. RCF+Ethos ($N = 4$) vs. GBMF+Ethos ($N = 4$) 2-tail T-test $p = 0.4267$. PLK4 – RCF+Vehicle ($N = 4$) vs. GBMF+Vehicle ($N = 4$) 2-tail T-test $p = 0.0031$. RCF+Ethos ($N = 4$) vs. GBMF+Ethos ($N = 4$) 2-tail T-test $p = 0.0498$. Representative experiments shown.

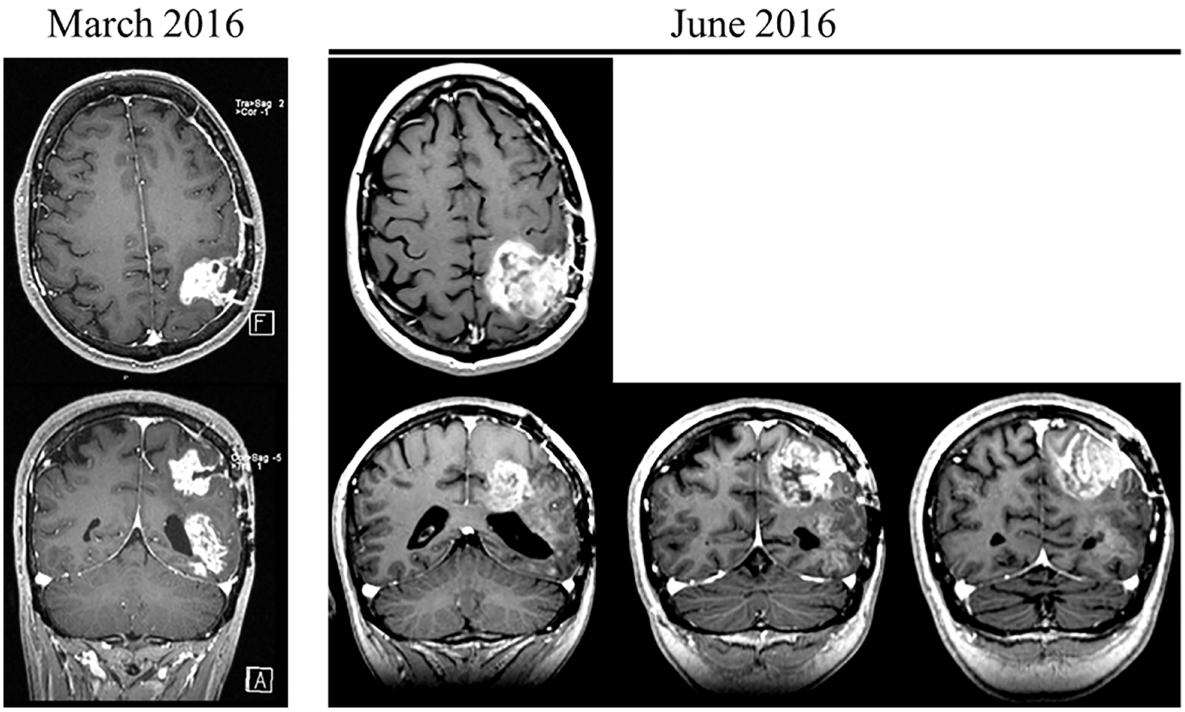

Supplementary Figure 7: Baseline and disease progression of a 38-year-old patient. Baseline imaging occurred March 2016 and progression of disease was noted on axial and coronal T1 post-contrast imaging in June 2016. Patient began compassionate treatment July 4th, 2016.

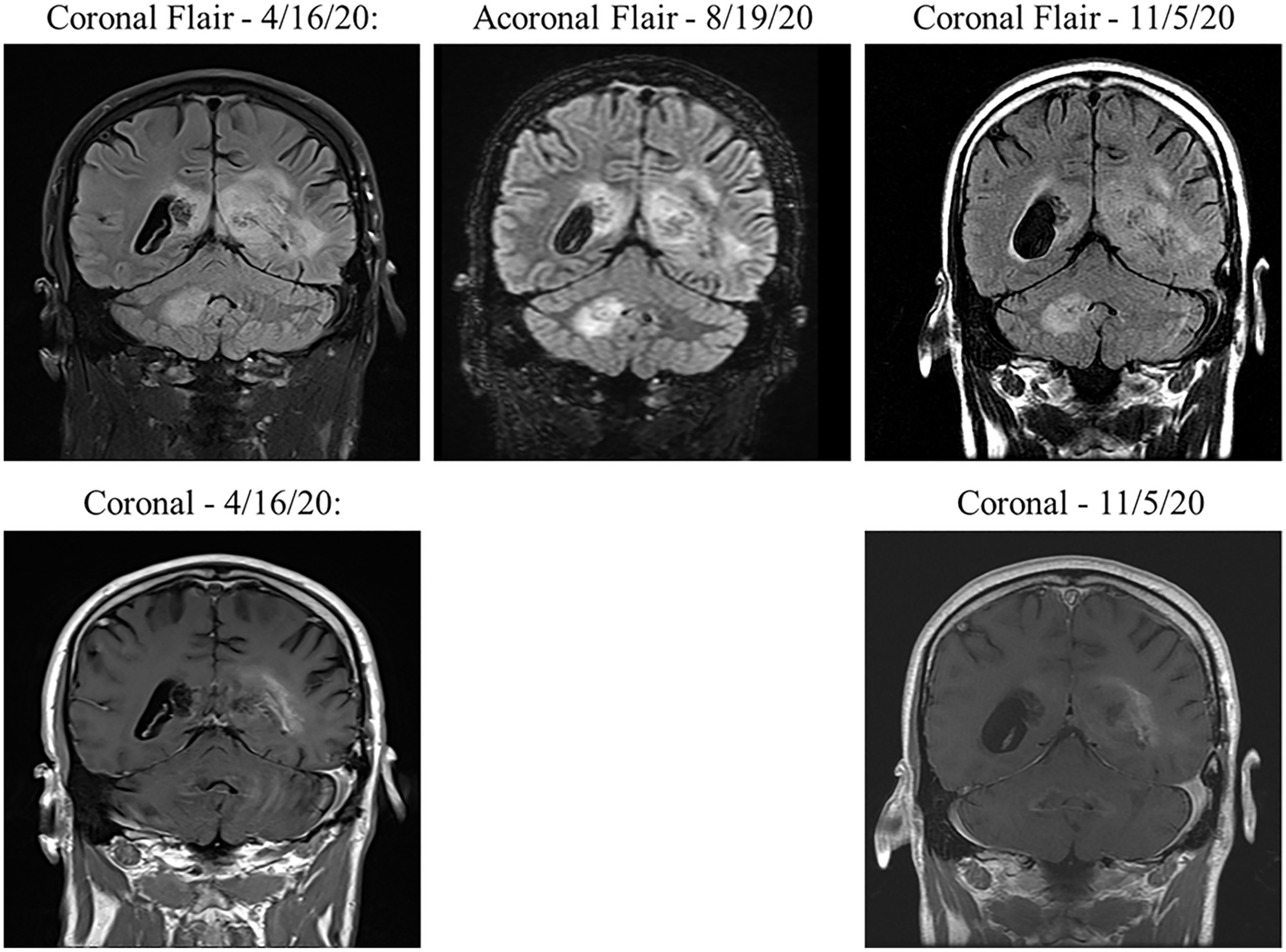

Supplementary Figure 8: MR images of a 47-year-old patient. 47-year-old male incidentally diagnosed with a left parietal brain tumor at the age of 32 following a fall. Following two years of monitoring, he underwent a left parietal craniotomy and subtotal resection of the lesion in 2007 which confirmed the presence of a grade II oligodendroglioma. Treatment with the TheraBionic device began on July 1st, 2020. MR images are two months post-treatment initiation and show stable disease. The patient was also on bevacizumab hence, the quality of the post-contrast scans negatively impacted as bevacizumab closes the blood brain barrier and dramatically reduces enhancement. April 16th, 2020 – image approximately six weeks before TheraBionic treatment, August 19th, 2020 – image during TheraBionic treatment, and November 5th, 2020 – image after discontinuing TheraBionic treatment. Top row: MR Fluid-attenuated inversion recovery (FLAIR) images used to assess infiltrating tumor and surrounding peritumoral edema. Bottom row: Coronal T1 post contrast images for the first (April 16th, 2020) and last studies (November 5th, 2020).
